# Internet use and psychological wellbeing among older adults in England: a difference-in-differences analysis over the COVID-19 pandemic

**DOI:** 10.1017/S0033291722003208

**Published:** 2022-09-30

**Authors:** Claryn S. J. Kung, Andrew Steptoe

**Affiliations:** Research Department of Epidemiology and Public Health, University College London, London, UK

**Keywords:** Barriers to Internet use, depression, digital divide, English Longitudinal Study of Ageing, loneliness, quasi-experimental study

## Abstract

**Background:**

Longitudinal evidence on how Internet use affects the psychological wellbeing of older adults has been mixed. As policymakers invest in efforts to reduce the digital divide, it is important to have robust evidence on whether encouraging Internet use among older adults is beneficial, or potentially detrimental, to their wellbeing.

**Methods:**

We observe depressive symptoms and loneliness of adults aged 50 + in the nationally representative English Longitudinal Study of Ageing, from before (2018/19) to during the coronavirus disease 2019 (COVID-19) pandemic (June/July and November/December 2020). Our quasi-experimental difference-in-differences strategy compares within-individual wellbeing changes between older adults who desired to use the Internet more but experienced barriers including lack of skills, access, and equipment, with regular Internet users who did not desire to use the Internet more. To reduce selection bias, we match both groups on demographic and socioeconomic characteristics that are predictive of Internet use. We assume that in the absence of COVID-19 – a period of increased reliance on the Internet – the wellbeing trajectories of both groups would have followed a common trend.

**Results:**

Compared with matched controls (*N* = 2983), participants reporting barriers to Internet use (*N* = 802) experienced a greater increase in the likelihood of depressive symptoms from before to during the pandemic, but not worse loneliness levels. This effect was stronger for women, those aged above 65 years, and those from lower-income households.

**Conclusions:**

Besides enabling access to digital services, efforts to ensure older adults continue to be engaged members of an increasingly digital society could deliver returns in terms of a buffer against psychological distress.

A number of studies have documented an increase in mental health problems in the population during the coronavirus disease 2019 (COVID-19) pandemic (Prati & Mancini, 2021), including among older adults (Oh et al., 2021). Due to ensuing lockdown restrictions and social distancing policies, the pandemic also marked a period of increased reliance on the Internet for various daily activities – including communication, leisure, work, and to obtain information and provisions. Therefore, of particular concern was that older adults facing barriers to Internet use were further worse off in terms of their psychological wellbeing.

Although the proportion of Internet non-users in the population has been on the decline over the past decade, the digital divide, where older generations are less likely than their younger counterparts to use the Internet, persists and continues to grow (Office for National Statistics, 2019). This divide became even more pressing during the pandemic, as older adults experiencing barriers to Internet use were less likely to be able to obtain high-quality information, engage with online services, or connect with other people (Watts, 2020).

We estimate the effects of barriers to Internet use – including lack of access, motivation, skills, and experience – on depressive symptoms and loneliness among older adults during the COVID-19 pandemic. Our findings contribute not only to the literature on key factors influencing psychological responses during the pandemic, which has implications for public health measures in future crises (Shevlin et al., 2021), but also more generally to past discussions on Internet use and wellbeing that were previously inconclusive (reviewed at https://osf.io/8sjtu).

The English Longitudinal Study of Ageing is a nationally representative sample of adults aged 50+ residing in private households in England. Beginning in 2002/03, interviews have been conducted biennially on their health, social, psychological, cognitive, and economic circumstances; we use data from the most recent interview prior to the pandemic in 2018/19 (Wave 9). During the pandemic, there were two additional waves of data collection (June/July 2020 and November/December 2020), which achieved a high response rate (74%), and was administered online or by telephone for those who were not able to respond online (17%).

Participants were asked ‘Would you like to be able to use the Internet more frequently or for more things?’ in June/July 2020. We define participants responding ‘Yes’ as experiencing barriers to Internet use; and define those responding ‘No’ and, in addition, used the Internet at least weekly in 2018/19, as our control group. Barriers included ‘IT skills are not good enough’ (reported by 59% of participants with barriers), ‘Don't trust the internet’ (34%), ‘No reason to use it more’ (26%), ‘Don't have access to good enough equipment’ (15%), and ‘Don't have good enough access to broadband’ (15%). Our outcomes are based on the Center for Epidemiologic Studies Depression (whether participants reported ⩾3 of 7 symptoms) and UCLA Loneliness scales (sum of 3 item ratings, with scores ranging from 3 to 9). These are further detailed at https://osf.io/8sjtu.

As participants experiencing barriers to Internet use could differ systematically from regular users, particularly in demographic and socioeconomic characteristics that are also related to Internet use among older adults (e.g. age, partnership status, education, income, neighbourhood deprivation; Hunsaker and Hargittai, 2018), we conduct a coarsened exact matching process to mitigate selection bias in our statistical inference (Iacus, King, & Porro, 2019). In our final matched sample (*N*_*with barriers to use*_ = 802, *N*_*regular users*_ = 2983), the groups show no observable differences. Sample descriptions and our matching strategy are delineated at https://osf.io/8sjtu.

Figure 1 shows the outcome averages for participants experiencing barriers to Internet use (solid trajectory) and participants using the Internet regularly (dashed trajectory) across our observation period, after the matching process. Prior to the pandemic, both groups showed common trends in depression and loneliness, with some small fluctuations. Since the outbreak, there were clear increases in depressive symptoms and loneliness levels, consistent with other studies (Oh et al., 2021; Zaninotto, Iob, Demakakos, & Steptoe, 2022). Our hypothesis is that these deteriorations in wellbeing during the pandemic would be worse among participants facing barriers to Internet use, compared with regular users.

**Fig. 1.**
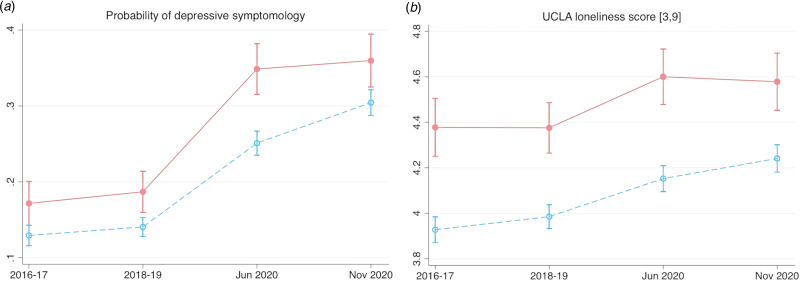
Wellbeing trajectories over time for the matched sample. The sample is restricted to those whose Internet use was observed at both 2018/19 and June/July 2020 waves, and for each outcome, further restricted to those for whom the outcome was observed at ELSA Wave 9 (2018/19) and at least one of the COVID-19 Waves. The solid trajectory represents older adults who desired to use the Internet more but experienced barriers to use (*N* = 802); the dashed trajectory represents regular Internet users who did not desire to use the Internet more (*N* = 2983).

We then compare individual-level changes in wellbeing from 2018/19 to June/July 2020 and November/December 2020 between the two groups, in a difference-in-differences estimation that eliminates all time-invariant confounders. We assume that in the absence of the pandemic, both groups would have followed a common time trend in wellbeing. Our estimation reveals that participants experiencing barriers to Internet use were 16.6 percentage points (95% CI 5.6–27.7) more likely to be experiencing depressive symptoms in June/July 2020 relative to 2018/19 (a 111% increase). This is significantly higher (*p* = 0.035) than the increase seen for regular Internet users, which was 12.0 percentage points (or an 80% increase relative to 2018/19, 95% CI 1.4–22.6). This finding corroborates previous longitudinal studies documenting Internet use as beneficial for depression (reviewed at https://osf.io/8sjtu). Further subgroup analyses reveal that barriers to Internet use were more detrimental in terms of depressive symptoms for women, adults aged over 65 years, and those with lower income; compared with their respective counterparts.

In contrast, changes in loneliness during the pandemic relative to 2018/19 were not significantly different between the groups. Contrary to the popular narrative that the pandemic drove up loneliness levels, longitudinal studies of older people have shown only small increases, if any (Zaninotto et al., 2022). It could be that the perceived barriers did not affect existing virtual contact that met minimum social needs; though this does not preclude that these barriers prevented an increase in Internet-based contact frequency or type (e.g. using different platforms). All difference-in-differences regression output are available at https://osf.io/8sjtu.

There is a host of benefits in encouraging Internet use among older adults, including promoting acceptance and participation in digital care services, which has experienced a rapid and widespread increase across numerous countries during the COVID-19 pandemic (Kinoshita et al., 2020). Our findings suggest that investments into digital skills courses for older adults – including those that enhance attitudes towards the Internet (e.g. increasing trust and understanding of types of use) – and providing financial support to improve access, can provide further returns in terms of preserving and promoting psychological wellbeing, even in a time of crisis.
